# A Novel Experimental and Modelling Strategy for Nanoparticle Toxicity Testing Enabling the Use of Small Quantities

**DOI:** 10.3390/ijerph14111348

**Published:** 2017-11-06

**Authors:** Marinda van Pomeren, Willie J. G. M. Peijnenburg, Nadja R. Brun, Martina G. Vijver

**Affiliations:** 1 Institute of Environmental Sciences (CML), Leiden University, P.O. Box 9518, 2300 RA Leiden, The Netherlands; willie.peijnenburg@rivm.nl (W.J.G.M.P.); n.r.brun@cml.leidenuniv.nl (N.R.B.); vijver@cml.leidenuniv.nl (M.G.V.); 2 National Institute of Public Health and the Environment, P.O. Box 1, 3720 BA Bilthoven, The Netherlands

**Keywords:** aquatic toxicology, zebrafish embryo, translational modeling, (metallic) nanoparticles, nano-specific testing, risk assessment

## Abstract

Metallic nanoparticles (NPs) differ from other metal forms with respect to their large surface to volume ratio and subsequent inherent reactivity. Each new modification to a nanoparticle alters the surface to volume ratio, fate and subsequently the toxicity of the particle. Newly-engineered NPs are commonly available only in low quantities whereas, in general, rather large amounts are needed for fate characterizations and effect studies. This challenge is especially relevant for those NPs that have low inherent toxicity combined with low bioavailability. Therefore, within our study, we developed new testing strategies that enable working with low quantities of NPs. The experimental testing method was tailor-made for NPs, whereas we also developed translational models based on different dose-metrics allowing to determine dose-response predictions for NPs. Both the experimental method and the predictive models were verified on the basis of experimental effect data collected using zebrafish embryos exposed to metallic NPs in a range of different chemical compositions and shapes. It was found that the variance in the effect data in the dose-response predictions was best explained by the minimal diameter of the NPs, whereas the data confirmed that the predictive model is widely applicable to soluble metallic NPs. The experimental and model approach developed in our study support the development of (eco)toxicity assays tailored to nano-specific features.

## 1. Introduction

Nowadays, the field of nanotechnology is accelerating in fabricating specifically engineered nanoparticles (NPs) which meet consumer needs. Economists predict, for the period from 2015 to 2020, an annual medial turnover of up to $3 trillion [1]. Next to first-generation NPs consisting of mono-elemental single sized nanomaterials, nowadays, complex nano-sized compounds, such as composites or oddly-shaped nanoparticles, are synthesized. For all these emerging NPs, information on fate and toxicity is vital knowledge to warrant the design of NPs that are safe for humans and the environment.

There are, nevertheless, concerns regarding NP-specific modifications needed for proper toxicity testing of NPs [2,3]. A first challenge with regard to NPs is that they often are synthesized in small quantities, especially in the design and testing phases of product development. Standard testing guidelines based on soluble chemicals, such as those prescribed by the OECD (Organization for Economic Co-operation and Development [4]), typically prescribe a vast amount of compound to be used in at least five test concentrations having twenty replicates. In the case of zebrafish embryo testing, a minimum of 2 mL exposure medium per replicate is needed in common practice (guideline 236, 2013 [4]). The exposure medium should be refreshed daily during the testing, whilst the maximum test duration is six days. This sums up to 240 mL of exposure suspension for each concentration to be tested and implies that, for instance, for testing a concentration of 1000 mg/L only (as often done in toxicity testing [5]), 240 mg of compound is needed. For a full range of five concentrations up to 1000 mg/L, this adds up to 720 mg of compound. On top of this, quite some additional material is needed for fate characterization and assessment of the physicochemical properties of the NPs. After all, not only the chemical fate of the NP should be determined, but also the colloidal and particle fate and behavior. Size determination of the colloids in solution (via dynamic light scattering assessment) commonly requires 10 mg of the NPs, including samples for transmission electron microscopy pictures. The measurements of the total concentration demands for a minimum of 25 mg of the NPs, followed by the same amount of the NPs needed to measure the ion concentration in the samples. This adds up to additional amount of at least 60 mg for chemical assessments. Overall, the amount of NPs needed to generate the very basic data regarding the fate and effect profile, strongly exceeds the quantities of material typically available in the initial research and development (R and D) phases. Hence, novel strategies are needed to reduce these amounts as much as possible.

The second challenge is the fact that debate is still ongoing regarding the question: which dose metric to use in order to properly express toxicity of NPs? Given the differences in size and shape of NPs and subsequent impacts of these properties on particle toxicity, it is unlikely that the mass of NPs administered is a proper descriptor of the actual dose causing toxicity [6,7].

The third challenge is that only rarely acute effects due to exposure to NPs are shown. Therefore, the determination of responses requires either availability of modified experimental protocols enabling to screen more sensitive sub-lethal endpoints, or increased modeling efforts that allow prediction of sub-lethal effects of hitherto non-tested NPs. With modeling effort, data gaps can be filled via interpolating approaches using similarity analysis [8] and eventually extrapolated between species via toxicity relationship assessments [9].

In view of these challenges, the aim of our study was to develop novel testing strategies that allow for efficient fate and toxicity assessments that require only small amounts of testing materials whilst achieving the same sensitivity with regard to the assessment of the endpoints of toxicity as in the corresponding standardized OECD tests. Previously, attempts were already made to reduce the amount of material needed [10,11], and these attempts are further improved in this study. Our study aimed to: (a) develop new experimental testing strategies enabling to work with low quantities of NPs; (b) determine the best dose-metric to describe the toxicity of particles, and (c) develop translational models for dose-response predictions for NPs even for those NPs that are classified as inducing low toxicity.

Experiments were conducted using three differently shaped Ag NPs. Since Ag NPs were found to display relative high rates of ion release, they are a clear example of non-stable metallic NPs. Ag NPs are classified as being toxic with LC_50_ values between 1 and 10 mg/L [12]. Experiments were also conducted with TiO_2_ NPs. These NPs are known to be chemically inert, thus allowing to test suspensions of stable metallic NPs as opposed to the tests performed with metallic NPs that dissolve during testing (non-stable NPs). TiO_2_ is classified to be harmful to the ecosystem (LC_50_ value between 10 and 100 mg/L [12]). By comparing the predictive power of the translational models for non-stable NPs with predictions made for stable metallic NPs, the applicability of the model to a wide range of NPs was tested. The results obtained for non-spherical NPs are discussed in view of the development of a regulatory framework to assess the safety of manufactured nanomaterials.

## 2. Materials and Methods

### 2.1. Preparation of Particle Suspensions

Spherical Ag NPs with nominal size of 20 nm (nominal surface to volume ratio 0.3) were purchased from SkySpring Nanomaterials, Inc. (Houston, TX, USA). A suspension of Ag nanoplates was obtained from Moscow State University (Moscow, Russia). Elongated Ag rods with a nominal size of 50 nm × 0.6 to 12 μm (nominal surface to volume ratio of 0.08) in suspension were purchased from Fraunhofer ISC (Würzburg, Germany). Spherical TiO_2_ NPs with a nominal size of 20 nm (nominal surface to volume ratio 0.3) were obtained from Io-Li-Tec nanomaterials (Heilbronn, Germany). Bipyramidal TiO_2_ NPs and TiO_2_ nanoplates were obtained from the Centre of BioNano Interaction (University College Dublin, Dublin, Ireland). The Ag nanospheres and nanoplates were coated with PVP (polyvinylpyrrolidone), and all other NPs were uncoated. Stock solutions were prepared by weighing dry powdered Ag or TiO_2_ particles and adding them into egg water (consisting of 60 μg/mL Instant Ocean Sea Salt, Sera Marin, in Milli-Q water, pH 6.5–7.0). Ag^+^ solutions were obtained by adding silver nitrate (AgNO_3_: CAS 7761-88-8, Sigma Aldrich, Zwijndrecht, The Netherlands) to egg water. All stock solutions were freshly prepared and sonicated for 10 min in an ultrasonic water bath (USC200T, VWR, Amsterdam, The Netherlands). Prepared stock suspensions or manufactured suspensions were diluted using egg water and embryos were exposed immediately after preparation.

### 2.2. Physicochemical Characterization

The size and morphology of the suspended Ag NPs and TiO_2_ NPs were characterized using transmission electron microscopy (TEM; JEOL 1010, JEOL Ltd., Tokyo, Japan) after 1 h of incubation in egg water. Dynamic light scattering assessments were performed on a Zetasizer Nano-ZS instrument (Malvern Instruments Ltd., Malvern, UK). The assessments allowed detection of the size distribution and the zeta-potential of Ag and TiO_2_ suspensions in egg water at 1 h and 24 h.

The concentrations of dissolved Ag ions and AgNP_(total)_ in egg water were analyzed using flame atomic absorption spectroscopy (AAS; Perkin Elmer 1100B, Waltham, MA, USA). Freshly-prepared dispersions (*t* = 0 h) and dispersions equilibrated for 24 h were used for this purpose. HCl (CAS 7647-01-0, Sigma Aldrich, Zwijndrecht, The Netherlands) was added to avoid loss of Ag due to precipitation and sorption to the wall of the vials. To determine concentrations of dissolved Ag ions in the particle suspensions, 7.5 mL of the supernatant was sampled after centrifugation of Ag NP suspensions at 5000× *g* for 10 min to remove NPs and ultimately determine the soluble silver fraction in the solution [13].

Titanium concentrations in the suspensions of nanoplates and nanobipyramids were analyzed following digestion with aqua regia (HCl: CAS 7647-01-0; HNO_3_: CAS 7697-37-2, Sigma Aldrich, Zwijndrecht, The Netherlands) and are determined using ICP-OES (Vista-MPX, Varian Inc., Santa Clara, CA, USA). The centrifugation step to determine ion concentrations in suspension was not included for TiO_2_ NPs because TiO_2_ particles do not undergo dissolution in aquatic suspensions [14]. Ti concentrations were measured immediately after preparation of the suspensions (0 h) and 24 h after preparation.

### 2.3. Experimental Setup

The OECD guideline 236 was modified as described in detail below. The modified experimental design uses in total roughly 1% of the amount of chemical required by the standard zebrafish embryo test protocol [4].

### 2.4. Zebrafish Husbandry

Zebrafish were handled as described by animal welfare regulations established in 2014 and they were maintained according to standard protocols (http://ZFIN.org). Adult zebrafish were maintained at 25 ± 5 °C in a 14 h light: 10 h dark cycle. Fertilized zebrafish eggs were obtained from an AB/TL wild-type zebrafish.

### 2.5. Toxicity Assay for Ag NPs

An acute exposure regime of 120 h was used, from 24 h post fertilization (hpf) to 144 hpf, thus including exposure during all major stages of embryonic development. The background mortality at 24 hpf was less than 10% (data not shown). The first adjustment to the OECD guideline is by following the protocol of Hua et al. [15], starting the exposure at 24 hpf. Thereafter, instead of transferring one embryo per well of a 24-well plate, 10 zebrafish embryos were distributed into each well of a 24-well plate in 2 mL of freshly prepared egg water containing a negative control, various concentrations of AgNO_3_ or various concentrations of Ag NP suspensions. One well was used per concentration. The nominal concentration range for the spherical NPs was 5 to 100 mg/L; the dilution factors for plates and elongated rods ranged from three to 30,000 times; the nominal concentration range for AgNO_3_ was 48 to 480 μg/L. Throughout the exposure, embryos and suspensions were kept at a temperature of 28 ± 0.8 °C. The exposure media were replaced with a freshly prepared suspension of NPs every day according to the OECD guideline 236 [4], except for days 4 (96 hpf) and 5 (120 hpf). The renewal procedure was shown not to increase NP concentrations over time [6]. Before each renewal, and at the end of the experiment, embryos were evaluated for morphological defects and death. All experiments were performed in triplicate. To verify the validity of our results obtained with the modified experimental protocol enabling to work with small amounts, we compared Cu NP data acquired with the modified test protocol to results obtained using existing methods as described by Hua et al. [15], see Supplementary Materials (Figure S1).

### 2.6. Toxicity Assay for TiO_2_ NP

TiO_2_ NPs were tested at a nominal concentration range from 10 to 1000 mg/L, using the same exposure conditions as described above for Ag NPs. The experiments with TiO_2_ NPs were performed under a commonly-used light-dark regime (14:10 h) and in addition using UV-light illumination using ultraviolet-a light (350 nm) with an intensity of approximately 1700 μW cm^−3^ for 14 h [16]. Temperature was maintained at 28 ± 0.8 °C during the experiments. All experiments were performed in triplicate.

### 2.7. Behavioral Analysis for TiO_2_ NPs

Before behavioral analysis, all living embryos (144 hpf) were evaluated in terms of normal development, morphological defects, and vitality using a stereo dissecting microscope. The behavioral analysis was performed by subjecting the embryos to the light–dark challenge test as modified according to Hua et al. [10]. Zebrafish embryos have a low locomotor activity under light exposure (basal phase). Sudden transition to dark induces a sharp spike of fast swimming activity lasting less than 2 s (challenge phase [10]). A total of 22 min of recording was used (Supplementary Materials, Figure S3): 10 min acclimatization, 4 min basal phase, 4 min challenge phase, and 4 min recovery phase. The total distance moved of each zebrafish embryo was tracked using the Zebrabox (Viewpoint, Lyon, France) and analyzed using VideoTrack software (Version 12, Viewpoint, Lyon, France).

### 2.8. Modeling

#### 2.8.1. Dose-Response Curves

Observational data of the fish embryos as obtained at 144 hpf were used to determine dose-response relationships—using mortality data and the sub-lethal malformation endpoints. For calculating lethal and sub-lethal effect concentrations, a sigmoidal dose-response model was used, available from the SPSS 23 software package (IBM, Armonk, NY, USA).

#### 2.8.2. Contribution to Toxicity of Ag Particles and Ions

An AgNO_3_ solution was used to quantify the toxicity of Ag ions to zebrafish embryos. This allowed us to determine the effect of the dissolved ion fraction in the solution (AgNP_ion_; as measured with AAS. The toxicity of the suspension (AgNP_total_) to zebrafish embryos was determined as being the sum of the response of the suspended particles (AgNP_particle_) and the response of the dissolved ions (AgNP_ion_). We applied the concept of response addition [17] as already used by Hua et al. [15] to compute the joint toxicity of metal ions shed from particles and nanoparticles. The response addition model is used because Ag ions and Ag NPs are assumed to elicit a response through different mechanisms. The model can be depicted as:

E_total_ = 1 − [(1 − E_ion_)(1 − E_particle_)] (1)

where E_total_, E_ion_, and E_particle_ represent the mortality of zebrafish embryos caused by the exposure to AgNP_total_, AgNP_ion_, and AgNP_particle_ (scaled from 0 to 1), respectively.

#### 2.8.3. Dose Metric Descriptors for the Translational Models

Dose metrics to be used within the dose-response modelling were chosen to be: minimal diameter, surface area, effective diameter, and surface to volume ratio. In each case, actual size information (obtained via TEM images) of the metallic NPs was used. When agglomeration occurred, identifiable single NPs present as commonly present on the surface of the agglomerates were used for size measurements. Only pristine sizes as derived from the TEM images were used for modeling, as is common for i.e., nano-QSAR (Quantitative Structure–Activity Relationship; [12]) and nano-QRA (quantitative read-across; [8]). For each dose metric, the parameter values were calculated based upon the diameter (d), length (l), and width (w) of the particles. For each metallic NP, multiple particles (*n* = 15 to 25) were measured and average values were used. The formulas used for calculating surface area and volume of differently shaped NPs can be found in the supplementary material, Table S1.

In order to use dose metrics in the translational models, we tested the following hypotheses: (i) NP toxicity increases with increasing total surface area; (ii) toxicity of the NP decreases upon increasing the smallest diameter as explained below (minimal diameter); (iii) toxicity decreases with increasing NP diameter calculated on the basis of the volume of the particles, independent of shape (effective diameter); and (iv) toxicity of NPs increases with increasing surface to volume ratio of the particles.

#### 2.8.4. Toxicity Prediction

Experimentally-obtained response data were plotted against the four dose metrics used. At first, the dose metric was expressed based on the total surface area of the particle [18], as based on the hypothesis that a higher total surface area represents more reactive surface area.

Secondly, for spherical and non-spherical particles, the dose metric was expressed as the minimal diameter in any dimension of the particle. It is hypothesized that the ability to penetrate into cells increases with decreasing minimal diameter [19,20].

Thirdly, the dose metric of spherical and non-spherical particles was determined by using the volume of the particle to calculate a fictional spherical diameter [21]. This effective diameter is reflected by the diameter of a spherical particle with the same volume. Here, too, it was assumed that NPs with small diameters have a higher ability to penetrate cells.

Fourthly, the dose metric was expressed as the surface to volume ratio of the NP. Both decreasing size and differences in particle shape modify the surface to volume ratio. Similar to total surface area, here an increasing ratio implies a larger reactive surface and, hence, increased toxicity [13].

For each parameter, a linear regression model was developed for the Ag NPs used in this study. Similar to the AgNP_particle_ data, secondary data [10,15,22,23,24] collected for AgNP_particle_, CuNP_particle_, NiNP_particle_, and ZnONP_particle_ were plotted as a function of the different dose metrics. The obtained linear dose metric relations for the non-stable metals Ag NP, Cu NP, Ni NP, and ZnO NP were used to calculate an overall linear regression coefficient ± standard deviation. Each regression line as obtained for the individual metallic NPs was given equal weight. In addition, a *p*-value was calculated indicating whether the initial slopes of the regression lines obtained for the Ag NPs, Cu NPs, Ni NPs and ZnO NPs differed significantly. Similarity of slope is assumed to reflect the appropriateness of a dose metric to predict toxicity across metallic NPs. Thereupon, the intercepts of the individual regression equations were calculated as these reflect the intrinsic reactivity of metallic NPs. Using the overall linear regression coefficients thus obtained, and based upon the limited availability of experimental data on TiO_2_ toxicity, the intercept of the dose metric relation of TiO_2_ NPs was calculated, after which LC_50_-values of TiO_2_ nanobipyramids and nanospheres were predicted.

#### 2.8.5. Statistical Analysis

Significant differences between the newly-developed and already-existing testing protocols [15] were tested using a two-tailed T-test. Data collected on the behavioral test were presented as mean ± standard error of the mean (SEM). The homogeneity of variance was checked using the SPSS 23 software package (Version 23, IBM, Amsterdam, The Netherlands). The significance level for all calculations was set at *p* < 0.05. Significant differences between the different exposures within each phase were tested using a one-way analyses of variance (ANOVA) with Tukey’s multiple comparison post-test. Dose-metric linear regression modeling was performed using Prism (Version 7, GraphPad, La Jolla, CA, USA), followed by comparison of the aligning of the regressions developed for the different metallic NPs (comparable with an ANCOVA), using the same software. The limit of significance was set at *p* < 0.01 to account for the low numbers of experimental data that were typically available for generating the regression lines.

## 3. Results

### 3.1. Physico-Chemical Characterization of Ag NPs and TiO_2_ NPs

#### 3.1.1. Transmission Electron Microscopy and Dynamic Light Scattering

**Figure 1:**
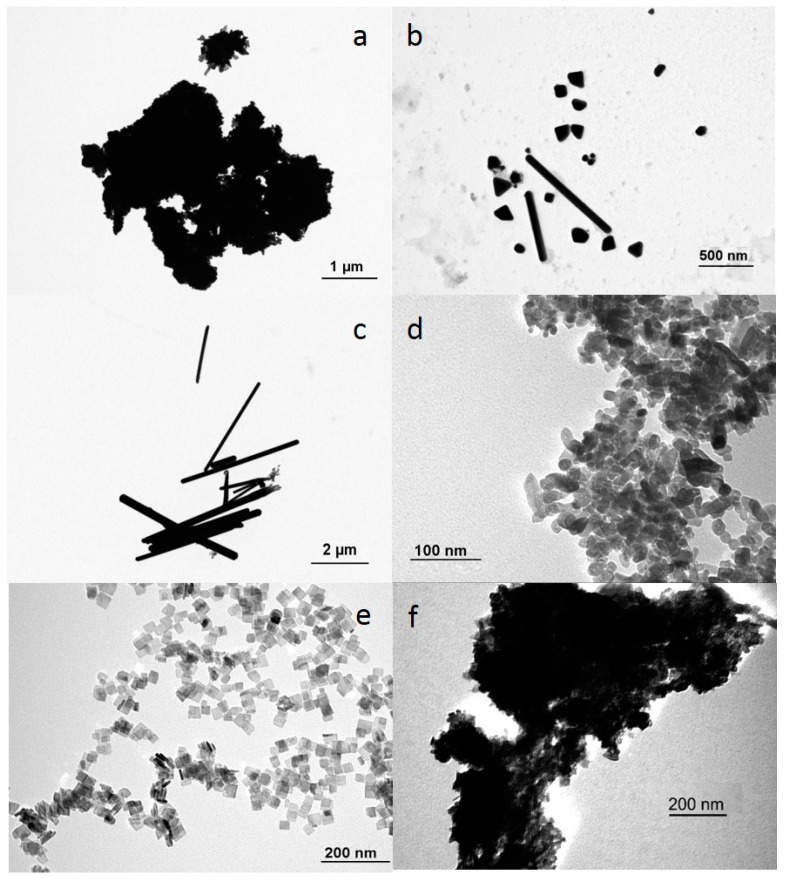
TEM images of (**a**) Ag nanospheres; (**b**) Ag nanoplates; (**c**) Ag elongated nanorods; (**d**) TiO_2_ nanobipyramids; (**e**) TiO_2_ nanoplates; and (**f**) TiO_2_ nanospheres.

TEM images showing size, shape, and clustering of the NPs after 1 h of incubation in egg water are given in Figure 1.

Large aggregates were formed immediately after the NPs were suspended in egg water. The PVP coated nanospheres aggregated to the largest extent. This general behavior was also evident from the size distribution patterns, with an average size of the Ag NP aggregates that was 43 times larger than the actual size of the individual NPs (Table S2, Supplementary Materials). PVP-coated Ag nanoplates responded differently and showed an aggregation size of only three times the actual individual NP size. Agglomerates of TiO_2_ NPs were even larger, with average agglomerate sizes being 56 times the size of the individual NPs. The zeta-potential of all NPs ranged between −30 to +0.6 mV over the test period and none of the zeta-potentials of the NPs changed significantly over time. Ag nanoplates appeared to contain Ag nanorods as well (see Figure 1b) in a number ratio of 10:90. Both shapes were, therefore, included into the calculation of the average surface to volume ratio.

#### 3.1.2. Metal Concentrations and Ion Release

As can be seen in Table 1, there was slight dissolution of Ag NPs after suspension. The amount of Ag ions released was related to the total concentrations measured (Table 1), and the shape of the Ag NPs was found to influence the extent of ion release. Nanoplates displayed the highest extent of dissolution, followed by nanospheres, whereas elongated nanorods released the lowest amount of Ag ions.

### 3.2. Toxicity Evaluation

Observations on mortality and developmental malformations of zebrafish embryos exposed to Ag NPs and TiO_2_ NPs were recorded. In addition, for TiO_2_ NPs exposures observations on behavioral movement (or swimming activity after light-dark challenge test) were also assessed and UV light was used to enlarge sub-lethal effects.

The results obtained using the modification of OECD test guideline 236, are given in Figure S1 of the Supplementary Materials. As can be deduced from this figure, implementation of this modification induced no statistically significant differences in toxicity between the original and the modified testing strategy (*p* > 0.05).

#### 3.2.1. Lethal and Sub-Lethal Effects of Ag NPs and TiO_2_ NPs

**Figure 2:**
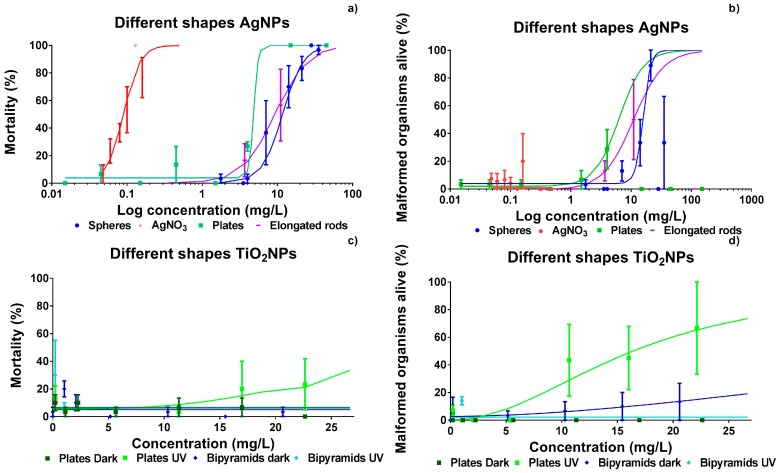
Dose–response curves for Ag NPs (**a**,**b**) and TiO_2_ NP (**c**,**d**) based on mortality and on number of malformed organisms. The dose is expressed as the log-transformed actual total concentration at T 0. Response data relate to 6 dpf embryos after days of exposure and are presented as means of three independent replicates ± standard error of the mean (SEM).

In Figure 2, the dose-response curves of Ag NPs and TiO_2_ NPs after five days of exposure (six days post fertilization; dpf) are displayed (lethality and malformations). The dose was calculated using actual total concentrations of suspensions of NPs at time 0 (T 0). Ag ions (Figure 2a,b), as tested using the AgNO_3_ solution, induced the highest toxicity with up to a factor of 50 higher toxicity in comparison to any of the Ag NPs suspensions tested. Ag nanospheres and elongated Ag nanorods displayed almost similar toxicity and spherically shaped Ag NPs were found to be the least toxic of all Ag NPs tested. Interestingly, when examining mortality (Figure 2a), Ag nanoplates were more toxic than elongated Ag nanorods. However, the nanospheres and elongated nanorods induced sub-lethal effects in up to 90% and 50% of the organisms, respectively, whereas the nanoplates and AgNO_3_ induced lower amounts of sub-lethal effects with 29% and 20% malformations, respectively (Figure 2b).

All LC_50_ and EC_50_ values were calculated based on the actual average AgNP_total_ and TiO_2_NP_total_ concentrations as measured at 0 h and after 24 h (Table S3, Supplementary Materials). In case of effects in between 20 and 50%, LC_50_ and EC_50_ values were predicted by extrapolation of the dose response curve. Since the effects observed for TiO_2_ NP bipyramids and nanospheres (data not shown) remained below 20% at all concentrations tested (Figure 2), full dose-response curves could not be assessed.

#### 3.2.2. Behavioral Assessment of TiO_2_ NPs

The results of the behavioral test using light-dark stress are displayed in Figure S2 (Supplementary Materials). In the behavioral test the total distance moved in mm is being used as a more sensitive sub-lethal endpoint compared to malformations. The results for embryos exposed to different concentrations of TiO_2_ NPs revealed lack of significant impact (*p* > 0.05 for all comparisons) of any of the TiO_2_ NPs tested.

#### 3.2.3. Relative Contribution to Toxicity of the Ag NP Particles and Ag Ions

In Table 2 the relative contribution to toxicity of the ions versus the particles is shown at the experimental LC_50_ levels of the suspensions tested. According to Table 2, most of the toxicity is induced by the particles, except for the Ag nanospheres. The EC_50_ levels are in the same range as the LC_50_ levels (Tables S4 and S5), which is related to the shape of the dose-effect curves. For AgNO_3_, only lethality was found. Hence, no EC_50_ value could be determined for Ag ions. Therefore, quantification of the relative contribution of Ag NPs for morphological responses is not possible.

### 3.3. Similarity Modeling to Estimate TiO_2_ NP Toxicity

**Figure 3:**
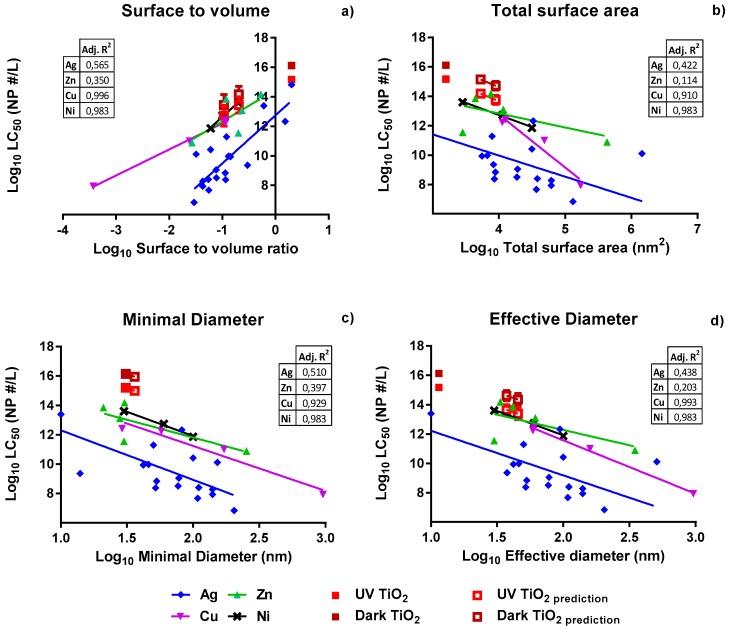
LC_50_ values of NP_particle_ (based on actual particle number concentrations at T 0) expressed using the following dose metrics: (**a**) surface to volume ratio; (**b**) total surface area; (**c**) minimal diameter; and (**d**) effective diameter. For each line the adjusted *R*^2^ is provided in the inserted table. Experimental LC_50_ values for TiO_2_ (filled squares) were calculated using SPSS. Predicted TiO_2_ values are shown by open squares with calculated standard error.

In order to predict the missing LC_50_ values (i.e., the effect levels for which even at the highest dose tested, no adverse response was recorded) of TiO_2_ nanospheres and nanobipyramids, similarity modeling was applied using the newly generated data and the collected secondary data. LC_50_ values of Ag NPs, Cu NPs, Ni NPs, ZnO NPs, and, where relevant, LC_50_ values calculated by SPSS (Version 23, IBM, Amsterdam, The Netherlands) for TiO_2_ NPs were expressed as particle numbers and plotted against the different dose metrics (Figure 3). Thereupon, predicted LC_50_ values for TiO_2_ nanospheres and nanobipyramids were plotted (Figure 3, open squares, the actual values are given in Table S4, Supplementary Materials). Reliability of these predictions was related to the predictive strength of each model. The predictive strength of each dose metric was assessed based upon the adjusted *R*^2^ values. The power of prediction decreased along the following dose-metrics: surface to volume ratio > minimal diameter > effective diameter > total surface area.

The average slopes and the corresponding *p*-values that are used to indicate the significance of the deviation of the slopes of Ag NPs, Cu NPs, Ni NPs, and ZnO NPs are given in Table 3. In comparison to the adjusted *R*^2^ values, the aligning of the slopes and their variance in intercepts changed the order in which the strongest predictive power is ranked to: minimal diameter > effective diameter > surface to volume ratio > total surface area. The minimal diameter, as well as the effective diameter showed the lowest variance of the slope across the various types of particles tested and resulted in significant differences in the values of the intercept of the regression lines (Table 3). This indicates the highest level of parallelism of the regression lines when using any of these two dose metrics to express toxicity. This highest level of parallelism in combination with the highest values of the adjusted *R*^2^ values depicted in Figure 3 was found for the case of the minimal diameter, indicating that the minimal diameter is the best descriptor of particle toxicity of metallic NPs like Ag, Cu, Ni, and ZnO, albeit with a marginal difference with the effective diameter. The differences in intercepts reflect differences in the intrinsic toxicity of metallic nanoparticles of similar volume.

## 4. Discussion

There are at least two challenges to overcome when modifying conventional ecotoxicity assays to comply with nano-specific needs. The first challenge that we identified is the observation that, in the case of newly-synthesized nanoparticles, often only small amounts of materials are available for toxicity testing and fate assessment. The other challenge is that the dose metric to express toxicity is still under debate, which hinders the development of predictive models.

### 4.1. Novel Experimental Setup

Dealing with small amounts of NPs for testing implies that the required test volume is a limiting factor in testing full dose-response relationships. In recent publications, adjustments to the OECD guideline 236 [4] were proposed using a 96-well plate in order to limit the amounts of test chemicals to be used [10,11]. Our experimental test setup described here could even reduce the total amount from 720 mL, as required within the OECD standard test, to 8 mL of exposure medium for each concentration by lowering the total test volume, combined with the addition of a higher number of embryos in a single well, without losing accuracy. In our case, this reduced the total amount of compound needed for the whole experiment (in triplicate) from 1900 mg to 66 mg. Although the setup of Lin et al. [11] achieved a similar reduction, their method was not suited for nanoparticles. Nanoparticles typically tend to sediment, and frequent renewal of the medium is required in order to maintain the experimental concentration as renewal reduces the particle loss due to sedimentation. Unfortunately, medium renewal is not included in the modification proposed by Lin et al. [11]. The quantitative deviations between the outcomes of the regular and the novel testing strategy were found to not differ significantly (*p* > 0.05; Figure S1, Supplementary Materials).

However, within the concentration range tested, response levels could not be determined for some of the nanoparticles (TiO_2_ nanospheres and nanobipyramids). In order to allow for testing of NPs with a low intrinsic toxicity, we used three differently-shaped TiO_2_ NPs, since TiO_2_ NPs are classified as being moderately harmful with LC_50_ values found to range between 10 and 100 mg/L [12]). The TiO_2_ nanospheres (data not shown) and nanobipyramids showed no effects even at the highest concentration tested (Figure 2). Additional UV-irradiation of the TiO_2_ NPs [5,14] increased the effects induced by nanoplates of TiO_2_, but this was not the case for the other two shapes. These two particles were not the only exception, since Faria et al. [25] also report extremely low toxicity of TiO_2_ particles in 8 dpf larvae which were exposed under a series of illumination intensities. Thereupon, these results are in line with the findings of Bar-Ilan et al. [23] on acute toxicity, reporting 50% mortality after chronic exposure of zebrafish during 12 days to suspensions containing 0.1 mg/L nanospheres having a diameter of 21 nm. The observation of a lack of morphological effects at early life stages of TiO_2_ NPs is supported by other authors [26,27] who report particle-dependent effects at the gene level only. This may eventually lead to reproductive effects [28,29].

In line with all previous studies on this topic [23,25,26,27,28,29], our results showed that actual concentrations were significantly lower for all tested NPs compared to nominal concentrations. Especially for TiO_2_ NPs, it is noteworthy to mention that the actual concentration was only 2–3% of the nominal concentration, hence, the amount of bioavailable NPs is much lower than the nominal concentration. This observation can be attributed to the agglomeration and sedimentation processes that occurred extremely rapidly during the experimental course (Table 1), as also reported by Bar-Ilan et al. [23]. Sedimentation of TiO_2_ NPs was reported to increase in suspensions of high ionic strength [30], which is the case in the exposure medium that we used for zebrafish embryo testing (0.853 M). Overall, these observations imply that the fraction of TiO_2_ NPs that is available for uptake via the water phase is extremely low. Subsequently assuming that zebrafish embryos are exposed via the water phase only, well explains the observed absence of toxicity.

### 4.2. Translational Modelling

The highest predictive power was obtained when using either the minimal or the effective diameter as a dose metric. Due to the shapes of the particles, the smallest diameter can be much smaller at a certain amount of particles per volume, compared to a spherical particle. Various studies [18,31] report that surface to volume ratio and total surface area [16] are proper dose metrics for various nanomaterials. Our results did not confirm these general findings, as surface to volume ratio was found not to be the best predictor of toxicity across NPs and as use of the total surface area actually yielded the lowest predictive power. This lack of correlation between surface area and responses was also seen by Wittmaack [32]. Instead, other dose metrics, including the number of particles and joint length (product of number of particles and mean size) were found to be more suited to this study [32]. It should be noted that our study included a variety of differently shaped NPs and, thus, offers a larger variety of surface areas for analysis, while other studies [18] covered spherical NPs only.

### 4.3. Toxicity Prediction

Our linear regression models were found to be good predictors for the toxicity of the metallic NPs Ag, Cu, Ni, and ZnO. Therefore, these models were used to calculate the effect levels for novel metallic NPs for which no adverse responses were observed even at the highest concentrations tested. In the suspensions used for toxicity testing of zebrafish embryos, no higher actual concentrations than 20.7 mg/L TiO_2_ NP for the nanobipyramids and 50.9 mg/L TiO_2_ NP for the nanospheres could be obtained. Based upon the model using the minimal particle diameter, all LC_50_ values for these TiO_2_ NPs are predicted to exceed 200.6 mg/L TiO_2_ NP, which is a concentration that is far above the maximum test concentration that we could achieve in the zebrafish medium employed in our study. Subsequently, these predictions nicely confirm the observed lack of effects.

The underlying reason as to why we were not able to observe any adverse effects in the case of testing of TiO_2_ NPs could be related to the fact that the mechanism of toxicity of TiO_2_ NPs, being stable metallic materials that do not release ions, differs from the mechanisms of toxicity of the labile metallic NPs tested in our study: Ag NPs, Cu NPs, Ni NPs, and ZnO NPs. The models developed for the labile metallic NPs are based on (acute) embryo mortality, whereas as stated previously, effects of TiO_2_ NPs were reported only after long-term exposure only [25,29], and restricted to the effects on gene expression [26,27] and reproduction [28,29], rather than morphological and lethal effects. To determine sub-lethal responses, we performed a light-dark challenge test on top of the TiO_2_ NP exposure + UV illumination. As discussed above, UV illumination generally enhances the reactivity of TiO_2_, hence, enlarging the potential toxicity. Moreover, the light-dark challenge test allows determining sub-lethal stress on stress responses. In our experiment, these additional assessments did not induce the responses that were observed by other researchers [25,29].

## 5. Conclusions

Fish embryo toxicity tests form an integral part of hazard identification within environmental risk assessment. To account for NP-specific issues to address in hazard testing of NPs, such as the availability of low amounts of testing material, novel experimental toxicity testing strategies are required. Our results show that modifications of the experimental setup assists in the development of testing approaches that allow applying smaller quantities of material. Toxicity was shown to be best described using the minimal particle diameter as a dose metric. A translational model could be developed on the basis of this dose metric that allows the prediction of effects for soluble metallic NPs. It is noteworthy that it is still a challenge to develop translational models for stable NPs that do not dissolve slowly. Given their low bioavailability, testing of stable NPs remains a challenge. Overall, it is to be concluded that translational modelling can assist in extrapolating the effects of non-stable metallic NPs towards effect prediction of stable NPs.

## Figures and Tables

**Table 1:** Actual concentrations of Ag NPs and TiO_2_ NPs in suspension at 0 and 24 h. For each dilution step, total concentration (NP_total_), dissolved ion concentration (NP_ion_) at 0 h, and total concentration (NP_total_) at 24 h are displayed for each NP tested.

Particles	Dilution	NP_total_ Concentration 0 h (mg/L)	NP_ion_ Concentration 0 h (mg/L)	NP_total_ Concentration 24 h (mg/L)
Ag nanospheres PVP coated	1	17.20	0.83	2.28
	2	11.40	0.17	2.04
	10	3.50	≤ 0.016	0.62
Ag nanoplates PVP coated	30	14.00	1.50	9.35
	100	0.06	≤ 0.016	0.02
	3000	≤ 0.016	≤ 0.016	0.09
Ag elongated nanorods	30	328.00	0.22	4.30
	100	1.21	0.028	0.10
	3000	0.41	0.028	0.19
TiO_2_ nanoplates	1	22.7	-	0.10
	10	2.17	-	0.09
	100	0.28	-	0.05
TiO_2_ nanobipyramids	1	20.7	-	0.08
	10	0.70	-	0.15
	100	0.18	-	0.08
TiO_2_ nanospheres	1	50.9	-	0.20
	10	-	-	-
	100	-	-	-

**Table 2:** Relative contribution (%) of AgNP_ion_ and AgNP_particle_ to toxicity at the LC_50_ (lethality) level. LC_50_ concentrations are presented as median concentration (95% confidence interval) and *n* = 3.

Particles	Median Concentration (mg Ag/L)	Relative Contribution to Toxicity (%)	
		NP_ion_	NP_particle_
LC_50_			
Ag ions	0.09 (0.08–0.10)	100	0
Nanospheres	11.7 (9.9–13.6)	100	0
Nanoplates	4.9 (4.8–5.0)	9.2	90.8
Nanorods	9.2 (5.7–12.7)	3.8	96.2

**Table 3:** Calculated average slope and corresponding standard deviation (SD) for effective diameter, surface to volume ratio, minimal diameter, and total surface area. For each parameter, the difference of the individual slopes of the metallic NPs is depicted by means of the *p*-value and the corresponding F-value in combination with the number of slopes (n), as well as the difference of the intercept (elevation) of the regression lines. For regression lines that are based on a maximum of five data points (see Figure 3), the significance level was set at *p* < 0.01.

Parameter	Average Slope	SD Slope	*n*	Slope		Intercept	
				*p*-Value	F	*p*-Value	F
Minimal diameter	−3.04	0.43	3	0.95	0.15	<0.0001	8.37
Effective diameter	−2.95	0.59	3	0.90	0.16	<0.0001	7.83
Surface to volume ratio	2.62	0.78	3	0.25	0.97	<0.0001	10.74
Total surface area	−1.91	1.17	3	0.49	0.73	0.0007	6.44

## Supplementary Materials

The following materials are available online at www.mdpi.com/1660-4601/14/11/1348/s1, Figure S1: Toxicity of 50 nm Cu NPs in 24- and 96-well plates, Figure S2: Behavioral performance in the light–dark challenge test, Figure S3: Example of light-dark challenge test recording, Table S1: Formulas for calculating the surface area and volume of NPs for different shapes, Table S2: Size, surface to volume ratio, and Zeta-potentials for each NP tested, Table S3: Overview of LC_50_ and EC_50_ values calculated for Ag NPs and TiO_2_ NPs, as well as the size and surface/volume ratio of the NPs, Table S4: Predicted particle number values and LC_50_ values for TiO_2_ nanospheres and nanobipyramids, Table S5: Overview of highest measured concentration, actual 50% effect concentrations, and predicted LC_50_ values.
