# Supplementary material for: A Novel Experimental and Modelling Strategy for Nanoparticle Toxicity Testing Enabling the Use of Small Quantities
Source: Int J Environ Res Public Health. 2017 Nov 6;14(11):1348. doi: 10.3390/ijerph14111348 (PMC5707987; doi:10.3390/ijerph14111348)
Supplement: Supplementary file 1 [file ijerph-14-01348-s001.pdf]

International Journal of
Environmental Research and Public Health

MDPI

Supplementary Materials: A Novel Experimental and Modelling Strategy for Nanoparticle Toxicity Testing Enabling the use of Small Quantities

Marinda van Pomeren, Willie J.G.M. Peijnenburg, Nadja R. Brun, Martina G. Vijver

9 pages, 3 figures, 5 tables

Contents

Figure S1. Toxicity of 50 nm Cu NPs in 24- and 96- well plates. Page S2

Figure S2. Behavioral performance in the light–dark challenge test. Page S2

Figure S3. Example of light-dark challenge test recording. Page S3

Table S1. Formulas for calculating the surface area and volume of NPs for different shapes. Page S3

Table S2. Size, surface to volume ratio, and Zeta-potentials for each NP Tested. Page S3

Table S3. Overview of LC_{50} and EC_{50} values calculated for AgNPs and TiO_2NPs, as well as the size and surface/volume ratio of the NPs. Page S4

Table S4. Predicted particle number values and LC_{50} values for TiO_2 nanospheres and nanobipyramids. Page S4

Table S5. Overview of highest measured concentration, actual 50% effect concentrations, and predicted LC_{50} values. Page S5

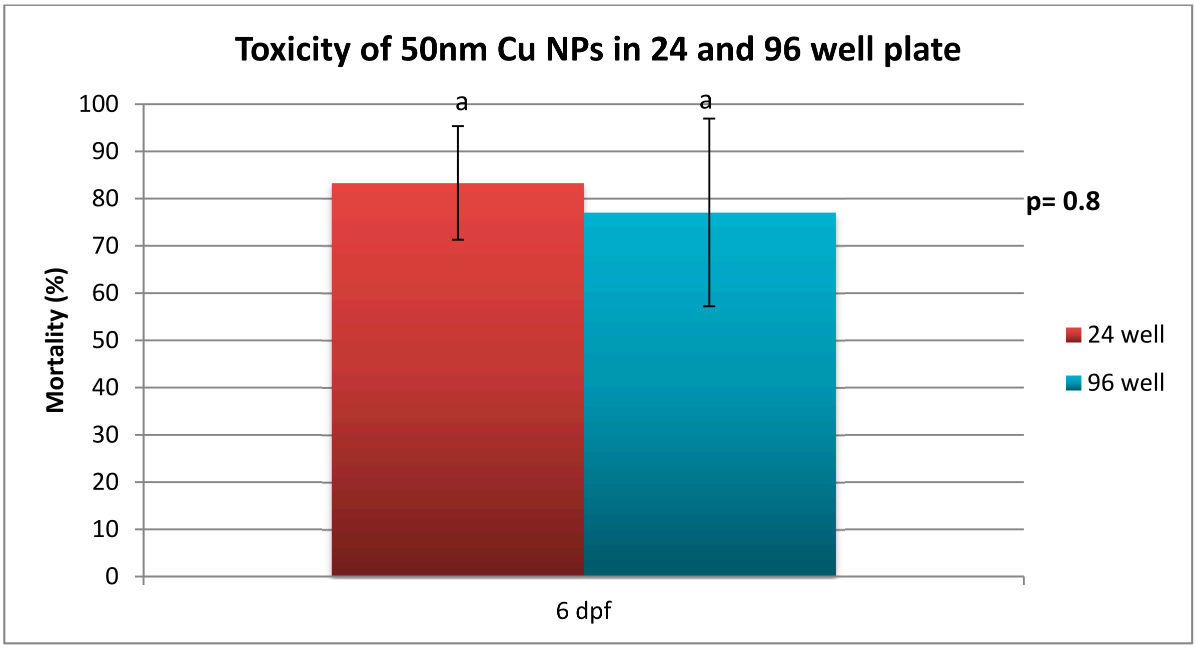

Figure S1. Comparison of two experimental designs: 24-well plate (novel method) and 96-well plate (as in [15]). The mortality rate of zebrafish embryos at 6 dpf exposed to 2 mg/L, 50 nm Cu. Given is the average ± SEM per exposure group and the *p*-value.

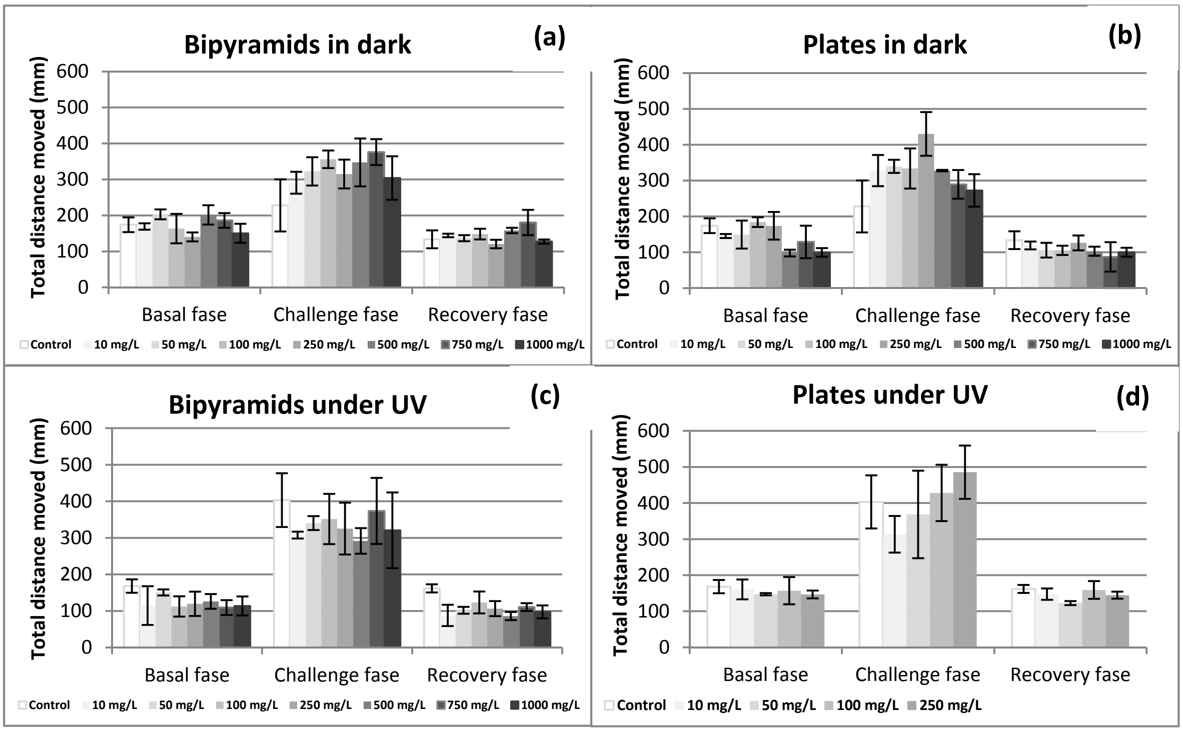

Figure S2. Behavioral performance in the light–dark challenge test. For each actual concentration of TiO_2 nanobipyramids (**a** and **c**) and nanoplates (**b** and **d**), the total distance moved was given in each phase. Set a and b were kept in the dark during the exposure period, set c and d were illuminated with UV during the exposure period. Data presented as mean ± standard error of the mean (SEM; n = 9).

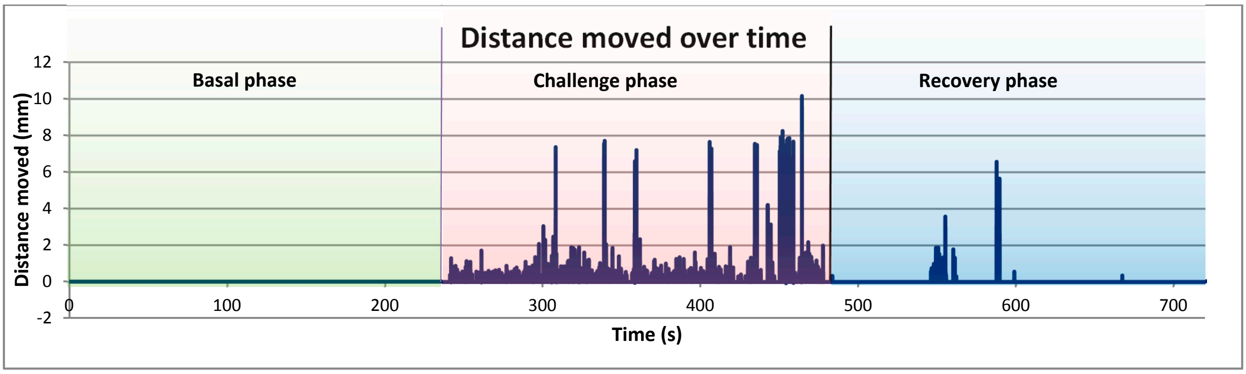

Figure S3. Example of light-dark challenge test recording of unexposed zebrafish embryos. In the figure, basal, challenge, and recovery phase are indicated.

Table S1. Formulas for calculating the surface area and volume of NPs for different shapes.

Shape	Surface Area	Volume
Spheres	$4\pi\left(\frac{d}{2}\right)^2$	$\frac{4}{3}\pi\left(\frac{d}{2}\right)^3$
Plates	$2lw$	lw
(Elongated) rods	$2\pi l\left(\frac{d}{2}\right) + 2\pi\left(\frac{d}{2}\right)^2$	$\pi\left(\frac{d}{2}\right)^2 l$
Bipyramids	8x side of pyramid= $8 * \left(\frac{1}{2}\, lw\right)$	2x pyramid $= 2 * (\frac{1}{2}\, lw)$

Table S2. Size, surface to volume ratio, and Zeta-potentials for each NP tested. Based on TEM images, the average diameter, length (L) and width (W) of the NPs are calculated, and subsequently used to calculate the surface/volume ratio.

Particles	Size Variation Using TEM (nm)	Surface/Volume Ratio	Size Distribution Using Dynamic Light Scattering (50% Percentile in nm)		Zeta-Potential Using Dynamic Light Scattering (mV)	
			1 h	24 h	1 h	24 h
Ag nanospheres	36.2 ± 19.7	0.23 ± 0.15	4339	996.1	−9.79	−9.91
Ag nanoplates	96.9 ± 43.9 (rods L 595.8 ± 471.0 W 73.9 ± 4.6)	0.85 ± 0.35	326.3	312.5	0.60	−1.20
Ag elongated nanorods	L 2712.0 ± 1726.6 W 148.9 ± 66.5	0.033 ± 0.013	7349	5352	−30.00	−26.60
TiO_2 nanoplates	28.3 ± 10.6	1 ± 0	2019	3945	−7.97	−11.90
TiO_2 nanobipyramids	L 68.2 ± 29.0 W 27.7 ± 13.1	0.20 ± 0.075	1934	2250	−3.51	−7.25
TiO_2 nanospheres	29.3 ± 37.4	0.24 ± 0.21	-	>1300	–	−20

Table S3. Overview of LC_{50} and EC_{50} values calculated for AgNPs and TiO_2 NPs, as well as the size and surface/volume ratio of the NPs. For TiO_2, effect levels are calculated for 'with UV' treatment and 'without UV' treatment. Toxicity values are calculated on the basis of the actual average concentrations at T 0 and at T 24, as can been found in Table 1. Note that the values indicated with an asterisk are calculated values due to the lack of actual LC_{50} and EC_{50} data.

Particles	Size Variation Using TEM	Surface/Volume Ratio	LC_{50} (95% Confidence Interval) (mg/L)		EC_{50} (95% Confidence Interval) (mg/L)	
			NP_{total} 0 h	NP_{total} 24 h	NP_{total} 0 h	NP_{total} 24 h
$AgNO_3$	-	-	0.09 (0.08–0.1)	0.09 (0.08–0.1)	na	na
Ag nanospheres	36.2 ± 19.7	0.23 ± 0.15	11.7 (9.9–13.6)	5.4 (4.5–6.2)	15.9 (14.0–17.8)	7.3 (6.4–8.2)
Ag nanoplates	96.9 ± 43.9 (rods<br>L 595.8 ± 471.0<br>W 73.9 ± 4.6)	0.85 ± 0.35	4.9 (4.8–5.0)	1.4 (1.3–1.5)	6.3 (1.4–11.3)	1.5 (1.2–1.9)
Ag elongated nanorods	L 2712.0 ± 1726.6<br>W 148.9 ± 66.5	0.033 ± 0.013	9.2 (5.7–12.7)	0.019 (0.018–0.021)	10.9 (4.0–17.8)	0.020 (0.017–0.023)
TiO_2 nanoplates with UV	28.3 ± 10.6	1 ± 0	35.8* (−14.1–85.9)	1.5* (−0.6–3.6)	16.4 (9.5–23.4)	0.10 (0.10–1.11)
without UV			394.5* (394.5–394.5)	16.4* (16.4–16.4)	na	na
TiO_2 nanobipyramids with UV	L 68.2 ± 29.0	0.20 ± 0.075	na	na	na	na
without UV	W 27.7 ± 13.1		na	na	64.2* (−170.2–298.5)	0.25* (0.24–0.27)
TiO_2 nanospheres with UV	29.3 ± 37.4	0.24 ± 0.21	na	na	na	na
without UV			na	na	na	na

Table S4. Predicted particle number values and LC_{50} values for TiO_2 nanospheres and nanobipyramids, calculated for four dose metrics.

TiO_2NP		Total surface area	Surface to volume ratio	Minimal diameter	Effective diameter
		**LC_{50} (Log_{10} Particle number #/L)**			
Nanosphere	Dark	15.1 (15.0–15.4)	13.4 (12.7–14.1)	15.9 (15.90–15.98)	14.4 (13.9–14.8)
	UV	14.2 (14.0–14.4)	12.7 (12.0–13.4)	15.0 (14.94–15.03)	13.4 (13.0–13.8)
Nanobipyramid	Dark	14.7 (14.5–15.0)	14.2 (13.6–14.7)	16.1 (16.14–16.16)	14.6 (14.3–15.0)
	UV	13.7 (13.5–14.1)	13.4 (12.9–14.0)	15.2 (15.19–15.20)	13.7 (13.3–14.0)
		**LC_{50} (mg/L)**			
Nanosphere	Dark	271.4 (190.3–493.3)	5.6 (1.1–28.7)	1826.1 (1660.1–2008.7)	47.3 (18.3–122.7)
	UV	30.0 (21.0–54.5)	1.1 (0.2–5.4)	201.6 (183.3–221.7)	5.2 (2.0–13.5)
Nanobipyramid	Dark	54.1 (32.5–127.7)	16.5 (4.7–58.8)	1605.8 (1580.4–1631.7)	46.8 (20.8–105.6)
	UV	6.0 (3.6–14.0)	3.1 (0.9–11.0)	177.3 (174.5–180.1)	5.2 (2.3–11.7)

Table S5. Overview of highest measured concentration, actual 50% effect concentrations (mortality and malformation), and predicted LC_{50} values, calculated for four dose metrics. Note that the values indicated with an asterisk are calculated values due to the lack of actual LC_{50} and EC_{50} data.

Particles	Highest NP_{total} Concentration 0 h (mg/L)	Actual Median Concentration (95% Confidence Interval) (mg/L)		Predicted LC_{50} (95% Confidence Interval) (mg/L)				Behavioral Effects (EC_{50})
		LC_{50}	EC_{50}	Total Surface Area	Surface to Volume Ratio	Minimal Diameter	Effective Diameter	
Ag nanospheres	17.20	11.7 (9.9–13.6)	15.9 (14.0–17.8)	-	-	-	-	-
Ag nanoplates	14.00	4.9 (4.8–5.0)	6.3 (1.4–11.3)	-	-	-	-	-
Ag elongated nanorods	328.00	9.2 (5.7–12.7)	10.9 (4.0–17.8)	-	-	-	-	-
TiO_2 nanoplates								
UV	22.7	35.8* (−14.1–85.9)	16.4 (9.5–23.4)	-	-	-	-	na
Without UV		394.5* (394.5–394.5)	na	-	-	-	-	na
TiO_2 nanobipyramids								
UV	20.7	na	na	6.0 (3.6–14.0)	3.1 (0.9–11.0)	177.3 (174.5–180.1)	5.2 (2.3–11.7)	na
Without UV		na	64.2* (−170.2–298.5)	54.1 (32.5–127.7)	16.5 (4.7–58.8)	1605.8 (1580.4–1631.7)	46.8 (20.8–105.6)	na
TiO_2 nanospheres								
UV	50.9	na	na	30.0 (21.0–54.5)	1.1 (0.2–5.4)	201.6 (183.3–221.7)	5.2 (2.0–13.5)	-
Without UV		na	na	271.4 (190.3–493.3)	5.6 (1.1–28.7)	1826.1 (1660.1–2008.7)	47.3 (18.3–122.7)	-

© 2017 by the authors. Licensee MDPI, Basel, Switzerland. This article is an open access article distributed under the terms and conditions of the Creative Commons Attribution (CC BY) license (http://creativecommons.org/licenses/by/4.0/).
